# Magnesium Oxide Nanoparticles: Dielectric Properties, Surface Functionalization and Improvement of Epoxy-Based Composites Insulating Properties

**DOI:** 10.3390/nano8060381

**Published:** 2018-05-30

**Authors:** Jaroslav Hornak, Pavel Trnka, Petr Kadlec, Ondřej Michal, Václav Mentlík, Pavol Šutta, Gergely Márk Csányi, Zoltán Ádám Tamus

**Affiliations:** 1 Department of Technologies and Measurement, Faculty of Electrical Engineering, University of West Bohemia, Univerzitní 8, 306 14 Pilsen, Czech Republic; pavel@ket.zcu.cz (P.T.); kadlecp6@ket.zcu.cz (P.K.); mionge@ket.zcu.cz (O.M.); mentlik@ket.zcu.cz (V.M.); 2 New Technologies-Research Centre, University of West Bohemia, Univerzitní 8, 306 14 Pilsen, Czech Republic; sutta@ntc.zcu.cz; 3 Department of Electric Power Engineering, Faculty of Electrical Engineering and Informatics, Budapest University of Technology of Economics, Egry J. Street 18., H-1111 Budapest, Hungary; csanyi.gergely@vet.bme.hu (G.M.C.); tamus.adam@vet.bme.hu (Z.A.T.)

**Keywords:** broadband dielectric spectroscopy, dielectric strength, loss factor, magnesium oxide, nanocomposite, relative permittivity, surface functionalization, voltage response

## Abstract

Composite insulation materials are an inseparable part of numerous electrical devices because of synergy effect between their individual parts. One of the main aims of the presented study is an introduction of the dielectric properties of nanoscale magnesium oxide powder via Broadband Dielectric Spectroscopy (BDS). These unique results present the behavior of relative permittivity and loss factor in frequency and temperature range. Following the current trends in the application of inorganic nanofillers, this article is complemented by the study of dielectric properties (dielectric strength, volume resistivity, dissipation factor and relative permittivity) of epoxy-based composites depending on the filler amount (0, 0.5, 0.75, 1 and 1.25 weight percent). These parameters are the most important for the design and development of the insulation systems. The X-ray diffraction patterns are presented for pure resin and resin with optimal filler amount (1 wt %), which was estimated according to measurement results. Magnesium oxide nanoparticles were also treated by addition of silane coupling agent (γ-Glycidoxypropyltrimethoxysilane), in the case of optimal filler loading (1 wt %) as well. Besides previously mentioned parameters, the effects of surface functionalization have been observed by two unique measurement and evaluation techniques which have never been used for this evaluation, i.e., reduced resorption curves (RRCs) and voltage response method (VR). These methods (developed in our departments), extend the possibilities of measurement of composite dielectric responses related to DC voltage application, allow the facile comparability of different materials and could be used for dispersion level evaluation. This fact has been confirmed by X-ray diffraction analyses.

## 1. Introduction

**Figure 1:**
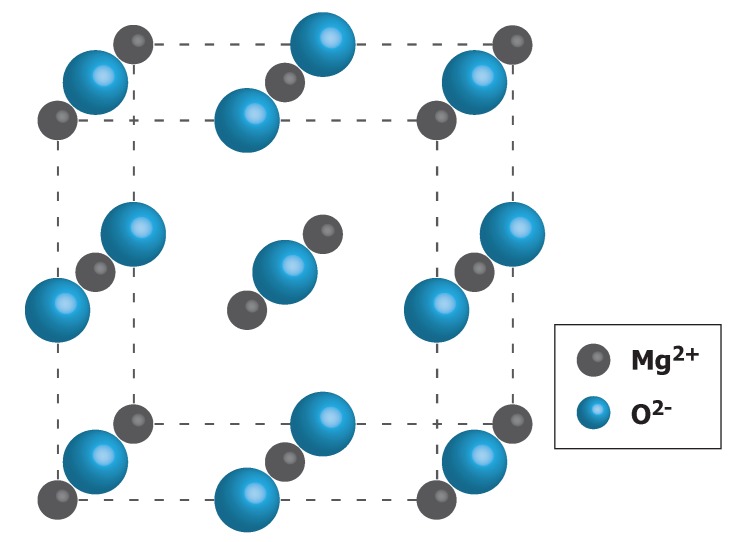
Structure of magnesium oxide crystal (Redrawn and adapted from [2]).

Magnesium oxide, often called periclase [1] (from Greek word periklao, peri—“around”, klao—“to cut”), is white hygroscopic solid mineral. Its empirical formula is MgO and its lattice consist of Mg${}^{2+}$ ions and O${}^{2-}$ ions, together bonded by ionic bond (Figure 1). Magnesium oxide is generally produced by the calcination of magnesium hydroxide Mg(OH)${}_{2}$ or magnesium carbonate MgCO${}_{3}$. Thermal treatment, used when calcination process occurs, affects the surface area and pore size and also the final reactivity of formed magnesium oxide. Used temperature can be divided into three groups, 700 ${}^{\circ }$C to 1000 ${}^{\circ }$C, where caustic calcined magnesium oxide is formed, 1000 ${}^{\circ }$C to 1500 ${}^{\circ }$C, where lower chemical activity magnesium oxide is formed and calcination over 1500 ${}^{\circ }$C, where reduced chemical activity type of refractory magnesium oxide is formed, that is mostly used for electrical and refractory applications [2].

Physical properties (see [3]) make magnesium oxide a good candidate for various applications. It is colorless to brown or black (based on the presence of iron or other foreign element). Considering the surface structure, it is visible that MgO has simplest oxide structure, called Rock-Salt structure. Its density is around 3.579 g/cm^3^ and hardness around 5 on Mohs scale. Thermal conductivity value of sintered magnesium oxide is defined at *T* = 100 ${}^{\circ }$C as 36 W/(mK). Due to refractory properties, the melting and also boiling points of magnesium oxide are very high (melting point: 2800 ${}^{\circ }$C, boiling point: 3600 ${}^{\circ }$C). Value of electrical resistance is depended on the purity of magnesium oxide. For high purity magnesia, the values of electrical resistivity can reach 10^16^ Ω·m. Specific resistance is mostly depended on chemical purity, but for higher values of temperature, i.e., 2000 ${}^{\circ }$C and more, the purity of magnesia does not have any influence on values of electrical resistivity. The dielectric constant of magnesium oxide is in the range from 3.2 to 9.8 at 25 ${}^{\circ }$C and under frequency 1 MHz, also values of dielectric loss for same conditions are around 10^−4^.

Chemical properties and surface composition of magnesium oxide are also influenced by the calcination procedure [4,5] (used temperature and used medium, i.e., air or vacuum) and also by the source of the precursor. Based on the various result, physical adsorption of water only occurs if MgO contains surface defects, such as high quantity of pores [6].

Applications of magnesium oxide includes various industry sectors. For their refractory properties, it is a valuable fireproofing ingredient in construction materials. Also in applications where corrosion [7] is not acceptable such as nuclear, chemical or superalloy industries. It has a usage in medical applications [8], where MgO is used for relief of heartburn and sour stomach, as an antacid, magnesium supplement, and as a short-term laxative. Other applications include insulators [9], fertilizers [10], water treatment [11], protective coating [12], etc.

Currently, there are trends to use nanoscale fillers [13]. In general, the nanotechnology is the production of functional structures in the range of 0.1–100 nm by various physical or chemical methods [14]. This fact also applies to magnesium oxide. The sol-gel technique [15] or hydrothermal technique [16] could be used for the production of nanoscale magnesium oxide. For an electrical application, e.g., in high voltage insulation, the MgO represents a prospective filler. Especially, due to wide band gap (7.8 eV) and high volume resistivity (10^17^ Ω·m). It is the highest value of volume resistivity from commonly used nanoscale oxides [17]. For this research, the MgO (supplied by NanoAmor [18]) with average diameter 20 nm, specific surface area more than 60 m^2^/g and density 0.3 g/cm^3^, was used.

A lot of studies have shown the effect of different types of nanofillers dispersed in epoxy-based composites on the mechanical [19,20], thermo-mechanical [21,22] or electrical [23] properties. The use of nanofillers has been also demonstrated to impove mechanical characteristics of many biomedical materials, mainly used in orthopedics [24] and dentistry [25]. However, this paper presents the unique results of nanoscale MgO dielectric properties itself, in the temperature and frequency range. These results are complemented by changes of dielectric properties of epoxy-based composites depending on the filler amount and by the effect of surface modification. These effects of surface functionalization were studied by two special measurement and evaluation techniques, i.e., reduced resorption curves (RRCs) [26] and voltage response method (VR) [27], which may be used as indirect method for evaluation of filler dispersion.

## 2. Dielectric Properties of MgO Nanoparticles via Broadband Dielectric Spectroscopy

Broadband Dielectric Spectroscopy (BDS) is a modern diagnostic method which allows interconnecting several measurement techniques to obtain a comprehensive view of the material behavior under an electric field with a frequency in very wide range. For this investigation, the main diagnostic unit of the Alpha-A measuring device (Novocontrol Technologies) has been used. It contains a frequency response analyzer with a sinusoidal signal generator and allows analyzing in the frequency range from 3 × 10^−6^ to 4 × 10^7^ Hz [28]. Used electrode system (ZGS type) is an active (incorporate a block for diagnostic signal processing) electrode system whose sample cell consists of two parallel cylindrical gold-plated electrodes and the tested flat sample is placed between these electrodes. The diameter of electrodes which are in direct contact with the sample (MgO pellet—see Section 2.1) is 10 mm and therefore the plate capacitor with a diameter of 10 mm is considered in calculations.

The aim of the BDS analysis is primarily to find out general trends of development (and some selected values) of dielectric constant and loss factor as results of measurement at variable frequency of the electric field and variable ambient temperature which is regulated by nitrogen vapor in the cryostat with inserted sample cell. Presented analysis was performed in the temperature range from 25 ${}^{\circ }$C to 150 ${}^{\circ }$C and in the frequency range from 0.5 Hz to 1 MHz. These ranges of set-up measurement parameters were chosen as sufficient with reference to the intended application of MgO as a filler for electrical insulating composites with a polymer matrix [29]. Entire measurement consists of two phases. The first phase called heating represents the period when the temperature in the cryostat was gradually increased from 150 ${}^{\circ }$C to 25 ${}^{\circ }$C with a step of 5 ${}^{\circ }$C. Then, the temperature was decreased from 150 ${}^{\circ }$C to 25 ${}^{\circ }$C with same temperature step in the next phase called cooling. The frequency of the measuring voltage with amplitude of 1 V was gradually decreased in the chosen range for each selected temperature in both phases [30]. Several tens of pair of $\epsilon^{\prime }$ and $\epsilon^{\prime \prime }$ values for different frequencies were obtained for each temperature (for heating and cooling) after the processing of measured data as a final result of dielectric analysis via BDS.

### 2.1. Preparation of MgO Pellet

Used electrode system as a part of the Broadband dielectric spectroscope do not enable measurement of a powder filler in the delivered state. Measurement is possible only with a sample with defined shape and dimensions. In particular, it is necessary to prepare a pellet with a structure, which is as homogeneous as possible, and with a defined thickness. Optimally prepared pellet can be placed between measuring electrodes without pellet fragmentation. The preparation of the pellet represents a homogenization of the MgO powder (more than 200 mg) in a ShakIR sample grinder (PIKE Technologies, Fitchburg, WI, USA) in the first step. The second step is a pressing of the powder whereas the amount of 200 ±1 mg of powder was loaded into the evacuable pellet press (PIKE Technologies) with a pressing chambers diameter of 13 mm. Air is evacuated from the pressing chamber during a compression. The evacuable pellet press is inserted between parallel pressing plates of the hydraulic press H-62 (Trystom, Olomouc, Czech Republic). It was necessary to optimize the maximum applied pressure in order to avoid a excessive deformation and a fixation of filler particles in pellet volume. This pressure was set to a value of approximately 340 MPa (force of 45 kN applied to anvils) to minimize the adhesion of the pellet surface to the anvils surface that may cause a damage of the pellet during removal from the chamber [31]. This pressure was determined on the basis of self-optimization of pellet preparation from MgO.

### 2.2. Comprehensive Analysis of Relative Permittivity

**Figure 2:**
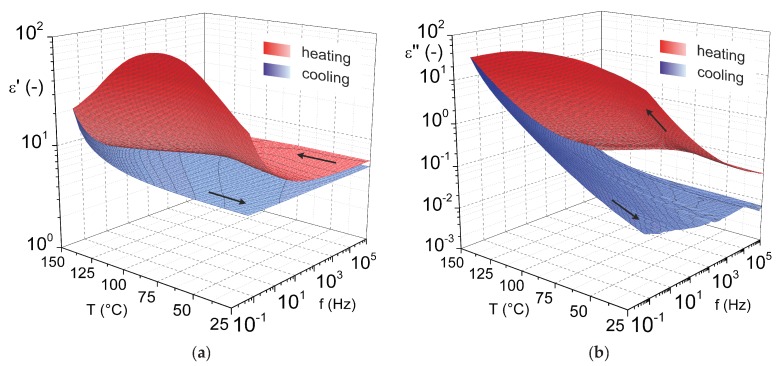
3D interpretation of frequency-temperature dependencies of (**a**) dielectric constant and (**b**) loss factor for heating (red scale) and cooling (blue scale) of MgO pellet.

The final result of dielectric analysis performed via BDS are interpreted primarily as complex 3D view (Origin^®^, OriginLab, Northampton, MA, USA) of frequency and temperature dependencies of dielectric constant and loss factor. These dependencies are shown in Figure 2, in which phases of the measurement under increasing and subsequently under decreasing temperature are distinguished in color.

The results of the analysis show significant increase in $\epsilon^{\prime }$ and $\epsilon^{\prime \prime }$ values with the decreasing of measuring voltage frequency and also with the increasing of temperature when the material is heated in the delivered state. This trend of $\epsilon^{\prime }$ and $\epsilon^{\prime \prime }$ development is visible for cooling too, but with a different character. The results for the heating also denote the formation of noticeably visible peak in the temperature dependencies of dielectric constant and loss factor. The 3D interpretation of results of cooling shows a significantly smoother surface without visible peaks. In general, lower values of $\epsilon^{\prime }$ and $\epsilon^{\prime \prime }$ are recorded always for cooling, whereas primarily the loss factors decline by several orders of magnitude compared with heating is detected for lower temperatures.

The increase of $\epsilon^{\prime }$ and $\epsilon^{\prime \prime }$ caused by the frequency decreasing is primarily the effect of the electrical conductivity of the tested material, which is commonly visible in the low frequencies area of 3D interpretation of similar analysis results. The general cause of the most pronounced increase of dielectric constant and loss factor in the highest temperatures area is a disordered thermal movement of particles in the MgO pellet which is become more apparent under increasing temperature not only for this material. The peak occurrence in characteristics is a result of changes in material structure which are caused by the temperature rise during the measurement and which significantly influence dielectric properties of MgO. Specifically, changes in structure are related to a process of MgO dehydration. This material evidently contains a significant amount of water molecules in the delivered state. However, these water molecules are with high probability only absorbed or very weakly bonded in the volume of MgO powder if they are released at temperatures below 100 ${}^{\circ }$C. The effect of dehydration of MgO (differences of $\epsilon^{\prime }$ and $\epsilon^{\prime \prime }$ between heating and cooling) is significant in the case of lower frequencies and mean temperatures which are the most important for the intended application in electrical engineering. Differences in values of dielectric constant and loss factor for selected temperatures and industrial frequency of 50 Hz are shown in Table 1. Results in this table prove the fact that the usage of MgO without dehydration in composites with thermoset matrix (with values of the loss factor lower by orders of magnitude than for MgO at evaluated temperatures) cured at room temperature can have a significantly negative effect on dielectric properties of the composite. On the other hand, after dehydration, MgO exhibits very similar or even lower values of the loss factor than the pure thermoset at evaluated temperatures.

## 3. Improvement of Epoxy Based Composites Insulating Properties

**Figure 3:**
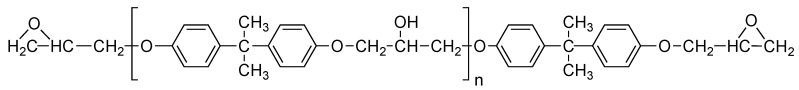
Structure of resin based on on Bisphenol-A diglycidyl ether.

The industrial epoxy resin (composition according to supplier safety sheet: Bisphenol-A and epichlorohydrin 50–70% (Figure 3); 1,4-Bis(2,3-epoxypropoxy)butane: 10–20%; Alkyl (C12–C14) glycidyl ether: 5–10%) with low processing viscosity and high bond strength was used for this experiment. This epoxy resin is curable at elevated temperatures (140 ${}^{\circ }$C, 4–6 h or 160 ${}^{\circ }$C 3–6 h) without additional hardener and it is commonly used in industry due to low viscosity and this is the main reason of our choice. Because lower viscosity of basic material ensures better dispersion of nanoparticles [32]. This resin is evaluated for thermal class H (IEC Standard 60085:2007 [33]), it is free of solvents and it is recommended for vacuum pressure impregnation (VPI) of rotating machines [34]. The density of selected epoxy resin is 1.12 g/cm^3^.

### 3.1. Production of Epoxy-Based Nanocomposites Samples

In this part, the sample production of pure epoxy resin and resin with dispersed nanoparticles will be described. Amount of 60 g of epoxy resin was used for the creation of a collection of the samples (5 pcs). In the case of pure epoxy, only magnetic stirring of the epoxy resin was carried out together with the vacuum venting (8 mbar) for 3 h. After this time, the epoxy resin was placed in the preheated Teflon molds with a silicone frame determining the height of the sample. The resin was then cured in a hot-air oven (140 ${}^{\circ }$C, 6 h). In case of matrices with dispersed particles, nanoparticles (0.5, 0.75, 1 and 1.25 wt %) were added to the already heated resin (75 ${}^{\circ }$C, 600 rpm, 3 h). An ultrasonic thorn was then used to break agglomerates (30 min) with simultaneous magnetic stirring and heating (70 ${}^{\circ }$C, 300 rpm). Further, the vacuuming (8 mbar) process was combined with magnetic stirring and simultaneous heating (90 ${}^{\circ }$C, 300 rpm, 3 h). The epoxy resin mixture was further placed in dried Teflon molds and cured under the same conditions as in the previous case.

### 3.2. Dielectric Properties of Epoxy-Based MgO Nano-Composites

**Figure 4:**
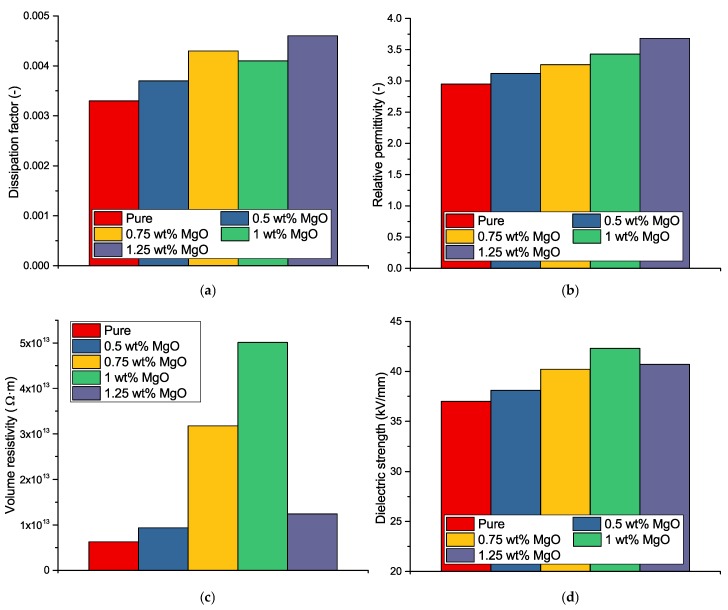
Changes of dielectric properties depending on the filler amount. (**a**) dissipation factor—500 V AC, 50 Hz; (**b**) relative permittivity—500 V AC, 50 Hz; (**c**) Volume resistivity—500 V DC; (**d**) Dielectric strength—increase 1.5 kV/s AC.

Four experimental measurements were performed for observation of MgO effect and finding the optimal filler ration to achieve the best dielectric properties in comparison with unfilled matrix (Figure 4). Namely dissipation factor and relative permittivity (IEC 60250:1969 [35]), dielectric strength (IEC 60243-1:2013 [36]) and volume resistivity (IEC 62631-3-2:2015 [37]) were measured according to mentioned standards. All measurements were performed according to Standard conditions given by IEC 60212:2010 [38]. Because of the basics of these parameters, no further information about step-by-step measurement procedure is provided in this article.

From presented results is visible that the addition of MgO nanofiller causes the changes of selected parameters. Possible reasons are discussed in following text. There is a slight increase of relative permittivity and dissipation factor, respectively. These parameters characterize the degree of polarizability of the matrix, filler and their interfaces and losses caused by their interactions with electric field. Different behavior can be explained by changing the curing reaction of the whole composite and by changing the degree of crosslinking [39].

Study [40] highlighted the positive effect of magnesium oxide of nanometric dimensions on the reduction of trapped charge in the internal structure of the material at a fill volume in the range 0.5–2%. Due to the nature of the particles, when their volume resistivity is in the order of 10^17^ Ω·m, the resistivity of whole composite may increased at low filling rates. This fact was confirmed by performed measurements. An increase of volume resistivity of the composites can be attributed to an increase in resistance to injection of the charge carriers and their generation in the internal dielectric structure [41]. Dielectric breakdown phenomena of nanodielectrics is affected mainly due to low quantity of agglomeration at low filler loadings [42]. Some studies [43,44] also shown the changes of relation between the dielectric breakdown and free-volume in polymers. In these cases, also a percolation threshold could plays a role, but not more works have been presented for percolation threshold estimation in the case of dispersed nanoscale insulating particles, where the character of added filler causes improvement of the electro-insulation properties of whole composite. On the other hand, the behavior of dispersed conductive fillers is very-well known [45,46,47,48] and percolation threshold could be estimated based on the significant increase of conductivity. However, the dielectric parameters of basic material could be improved by addition of relatively low amount of nanofiller, as is evident from our previous studies [49,50,51]. In connection with these claims, the study [52] describes a theory of percolation and interfacial characterisation via breakdown voltage measurement. It may be also used in this case for the confirmation of presented results (Figure 4d). In the case of pure epoxy resin, deeper traps generally exist. It results in relatively easy charge capture. If the charge carriers are released, the breakdown occurs due to their energy. The increase of breakdown voltage can be attributed to increasing of shallow traps inside the material by addition of nanofiller up to percolation threshold. The expected decrease will occur if the double-layers [53] on the particles surface are overlapped. It leads to the easier movement of the charge carriers in double-layers. Due to this fact, the conductive path will be formed [54] and breakdown can occur more easily. These effects are better noticable on the values of volume resistivity (Figure 4c), which goes hand in hand with the breakdown voltage measurement. According to the presented results, the percolation threshold could be estimated greater than 1 wt %.

Taking into account the preliminary measurements of the basic electrical properties (Figure 4), especially the volume resistivity and breakdown voltage, together with the the above-presented data, the optimal weight ratio for further investigation was set to 1 wt %.

### 3.3. X-ray Diffraction of Epoxy Resin and Epoxy Resin with Dispersed MgO Nanoparticles

**Figure 5:**
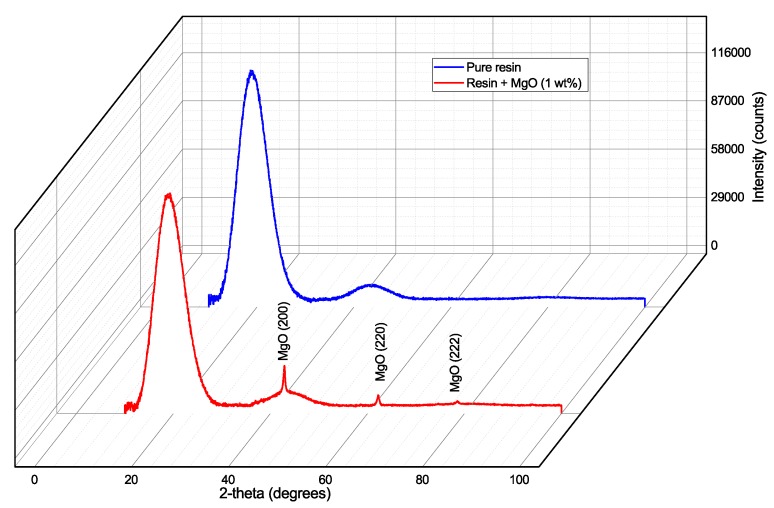
X-ray diffraction pattern of investigated materials.

The X-ray difraction has been used for the characterisation of internal structure of the tested material. These measurements were performed on a Panalytical X’Pert Pro (Malvern Panalytical) automated powder X-ray diffractometer using an X-ray lamp (IKa1 = 0.154 nm, 40 kV, 30 mA) and a semiconductor ultra-rapid PIXcel detector in the geometric Bragg-Bretan arrangement. The results from the diffractometer were aligned with the Pearson VII curves. From the X-ray diffraction analysis is visible the character of the amorphous material. This symbolizes very wide diffraction (6–8 degrees in the diffraction angles 2-theta), which are shown in Figure 5.

These diffractions, resp. their positions on the x-axis, corresponding with the results of presented studies [55,56]. On this diffractogram, only the diffraction pattern of MgO (200), (220) and (222) are noticed. Other lines are weak, or we do not notice them at all. Analysis of the profile of diffraction lines (200) showed that the size of the coherent dispersion region of X-ray crystallization (crystallite) is in all cases about 23–25 nm and the micro deformation is relatively low (0.0022–0.0025).

## 4. Surface Functionalization and Effect on Dielectric Properties

With regard to the fact that the fillers used for electro-technical applications are in more cases of inorganic origin, it is very difficult to achieve sufficient dispersion in the organic matrix under normal conditions [57]. One possible solution is the use of silane-based coupling agents. The most popular ones are γ-Glycidyloxypropyltrimethoxysilane (GLYMO) and γ-Aminopropyltriethoxysilane (APTES) which provide covalent interface links to prevent a phase separation [58].

A lot of studies have shown the effect of functionalization on different material properties. The surface of inorganic particles [59], glass fibers [60] and also the natural fibers e.g., jute fibers [61] or hemp fibers [62] have been already modified by addition of GLYMO or APTES, respectively. In general, the formula of silane coupling agent can be written as R(CH_2_)nSiX_3_ where the silane molecule is silicon (Si) and two functional substituents (R, X) that provide a bonding effect between the inorganic filler and the organic matrix [63]. The substituent X represents hydrolyzable groups (e.g., methoxy, ethoxy, alkoxy), and R represents an organofunctional group attached to the silicon atom by a hydrolytically stable bond. Most of the coupling agents comprise three hydrolyzable groups X and one organofunctional group R [64]. Coupling agents and their linear formulas are shown in Table 2.

**Figure 6:**
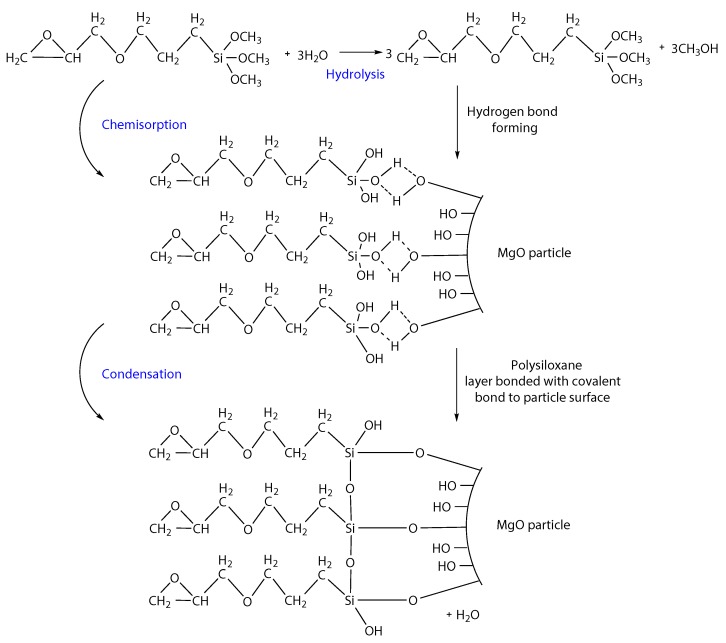
Simplified illustration of reaction of γ-Glycidoxypropyltrimethoxysilane with magnesium oxide surface (Redrawn and adepted from: [71,72,73]).

The reaction of the γ-Glycidoxypropyltrimethoxysilane with the magnesium oxide filler can be explained as follow and is illustrated in Figure 6. The corresponding silanol molecules are formed after hydrolysis of the hydrolysable groups [68]. Furthermore, the process of chemisorption is going. The hydrogen bonds are formed between silanol and –OH groups on the surface of magnesium oxide. A polysiloxane layer bonded with covalent bonds to the surface of the magnesium oxide is formed while water is released due to the condensation reaction [69,70].

The determination of the correct ratio [67] of the coupling agent can be based on the relationship (1)

(1) $$\begin{array}{cc} X & =\frac{A}{w}\cdot f, \end{array}$$

where *X* (g) is the amount of coupling agent to form the minimum cover layer, *A* (m^2^/g) is the specific surface area of the nanoparticle, *w* (m^2^/g) is the wetting specific area of the coupling agent and *f* (g) is the weight of nanoparticles. Formic acid or hydrofluoric acid may be applied first to the nanoparticle to increase the electro-kinetic potential of its surface [74]. For the ability to react with different types of matrices, γ-Glycidoxypropyltrimethoxysilane was chosen for this experiment. The density of the selected coupling agent is 1.07 g/cm^3^ and the wetting specific area is 331 m^2^/g. It is an epoxysilane coupling agent, in particular, an organofunctional trialkoxysilane having a high reactivity between epoxide rings and amino groups [63]. The amount of GLYMO was determined as 18.12% from the total weight of nanoparticles according to Equation (1) and above-mentioned parameters.

### 4.1. Production of Epoxy-Based Nano-Composites Samples with Treated Surface of MgO Filler

In this case, the dried nanoparticles (1% of total weight of epoxy resin) were first added to a solution of 96% ethanol 4% H_2_O (10 mL) and ultrasonic mixed (30 min). The coupling agent GLYMO (18.2% of total weight of nanoparticles) was then added to the mixture with re-application of ultrasonic mixing (2 h). Treated nanoparticles were added to already heated resin. The following procedure is the same as in the previous case.

### 4.2. Dielectric Properties of Epoxy-Based Nanocomposites with Treated Surface of MgO Filler

For comparison of the effect of the surface treatment, the measurement of dissipation factor (500 V AC, 50 Hz), relative permittivity (500 V AC, 50 Hz), volume resistivity (500 V DC) and dielectric strength (increase 1.5 kV/s AC) were repeated. The average values are shown in Table 3.

From the measurement results, it is clearly visible that the addition of coupling agent improved dielectric properties of the whole composite in comparison previous case. The relative permittivity was reduced approx. by 8%, due to the surface modification. The lower value of relative permittivity also guaranties a lower level of local stress inside the electrical insulation system. For example, in the case of an imperfect technological process during manufacturing of the insulating system of electrical machines and equipment. Mentioned decrease of relative permittivity could be caused by a changes of the degree of crosslinking due to the reaction of polymeric groups. They react with the coupling agent molecules on the nanoparticle surface and form a linear polymer chains in the interphase region [75]. The results further show that the dissipation factor of the composite is not negatively affected by the addition of the coupling agent, as confirmed other studies [39,75], as well. Addition of the coupling agent results in higher volume resistivity values, which can be attributed to a higher degree of the filler dispersion in the matrix and also to an increase of the energy levels of the electron traps [57], which results in a higher resistance to charge accumulation in the inner structure of the material.

### 4.3. Dielectric Response Measurement

Different optical methods and measurement techniques [76,77,78,79] are used for evaluation of the surface treatment effect or dispersion level, respectively. However, the idea of this paper is to evaluate the effect of surface treatment and particle dispersion by measurement of dielectric responses by special measurement techniques, i.e., reduced resorption curves (RRCs) and voltage response method (VR), which evaluate the conditions of the dielectric materials during charging and discharging process.

#### 4.3.1. Reduced Resorption Curves Analyses

**Figure 7:**
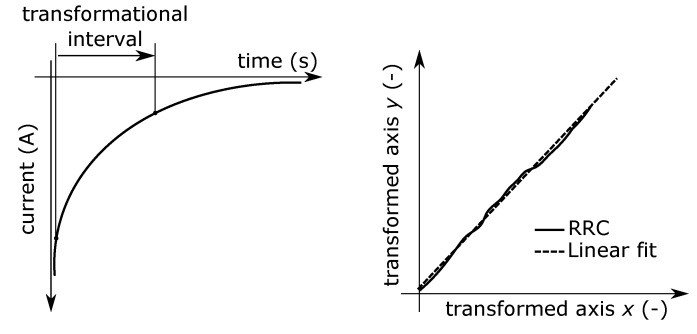
Transformation of resorption current to RRCs.

Dielectric absorption is a non-stationary phenomenon in dielectric materials after dc voltage application. Dielectric material is not able to follow the step change of the applied voltage. It means that the dielectric is charged for a certain time interval which is given by the relaxation time. This also applies to the discharging phenomenon. Both effects are caused by slow polarizations [80]. Here, the attention of our investigation was focused on the resorption characteristics. Resorption current can be used for reduced resorption curves (RRC) [26] determination. This method is based on the mathematical processing of time variable resorption current, which is transformed to relative resorption characteristic. The mathematical process of this methodology is expressed by Equations (2) and (3)

(2) $$\begin{array}{cc} x & =\ln(t)-\ln(15), \end{array}$$

(3) $$\begin{array}{cc} y & =\ln\left[ABS\left(i_{t}\right)\right]-\ln\left[ABS\left(i_{15}\right)\right], \end{array}$$

where *x, y* are transformed axes (-), *t* (s) is time, *i_t_* (A) is current in time*t, i*_15_ (A) is current in 15th seconds. The main parameter for this investigation is the slope of the linear fit from transformed data. The higher slope of the curve generally means better resistance to charge trapping. The adequate interval length is important for appropriate linear fitting. In general, the interval between 15 and 300 s is usable for this determination. This procedure is illustrated in Figure 7.

**Figure 8:**
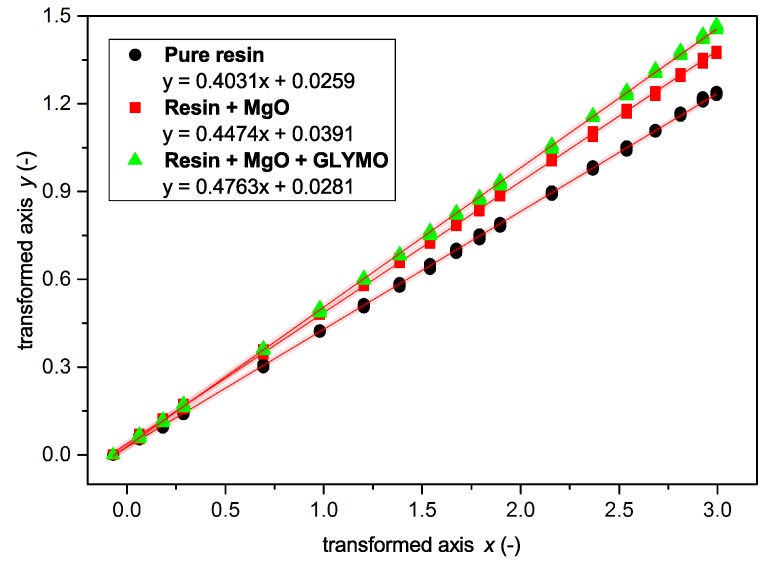
Reduced resorption curves for individual sample sets.

The electrometer KEITHLEY 6517A with suitable electrode system KEITHLEY 8009 Resistivity fixture was used for this measurement. The flat samples were conditioned (25 ${}^{\circ }$C, 35% RH) and short-circuited in shielding room for 24 h before the measurement. After that, samples were charged by DC voltage 1000 V for 3600 s. After charging, the resorption current was measured up to 600 s and was recorded by the developed script in VEE Pro software. Transformation interval was set in the range 15…300 s. Average values of resorption currents were transformed to reduced resorption curves (RRCs) according to Equations (2) and (3). The RRCs are compared in Figure 8.

From the results is clearly visible the slope increase of the trendline due to addition of filler resp. filler and coupling agent. In the first case, the slope increase may be caused by the reduction of bulk charge accumulation due to better resistance to charge injection and ionic carriers generation in the bulk of dielectric [41]. The increase of the slope in the case of addition of silane coupling agent is caused by better dispersion and by a higher level of miscibility between organic matrix and an inorganic filler. The other reason is the increase of the trap depth which may contribute to charge recombination [81]. It means that the charge suppression is more effective. From this point of view, there is the higher ability of the material to discharge the charge accumulated in the inner structure after dc voltage charging, because a higher level of energy is needed for its trapping.

#### 4.3.2. Voltage Response Analyses

**Figure 9:**
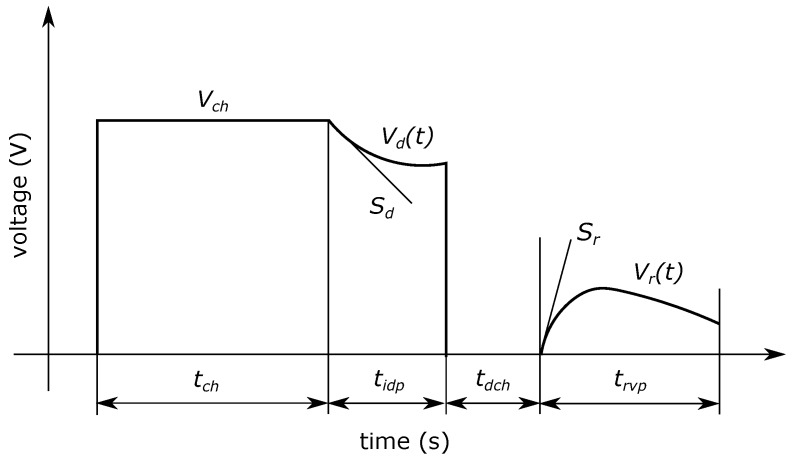
Timing diagram of Voltage Response measurement.

Originally, the Voltage Response measurement method (VR) was developed for investigation of oil-paper insulated cables and measures the initial slopes of the decay and return voltages [27]. The timing diagram of the measurement can be seen in Figure 9.

After a long duration (100…1000 s) charging period (*t*_*ch*_) the discharge voltage (*V_d_*(*t*)) is measured on the insulation for *t_idp_* time (<0.5 s). After a few seconds of short-circuiting (*t_dch_*) return voltage (*V*_*r*_(*t*)) is measured on charged insulation for *t*_*rvp*_ time (0.1…2 s).

The initial slope of *V*_*d*_(*t*) (marked with *S*_*d*_) is directly proportional to the conductivity of the insulation and the initial slope of *V*_*r*_(*t*) (marked with *S_r_*) is directly proportional to the polarization conductivity, in other words to the intensity of the slow polarization processes. Therefore, the separate investigation of conductive and polarization processes is ensured by the measurement of *S_d_* and *S_r_* since they have the same information content as *I_c_* conductive and *I_p_* polarization component of the leakage current, respectively [82]. The measured values can be seen in Table 4.

According to the measurement results, the pure resin had the highest conductivity and polarization conductivity from all the samples. By adding MgO filler, the conductive and polarization processes decreased significantly. However, the best results were measured after the addition of the silane coupling agent.

### 4.4. X-ray Diffraction of Epoxy-Based Nanocomposites with Treated Surface of MgO Filler

**Figure 10:**
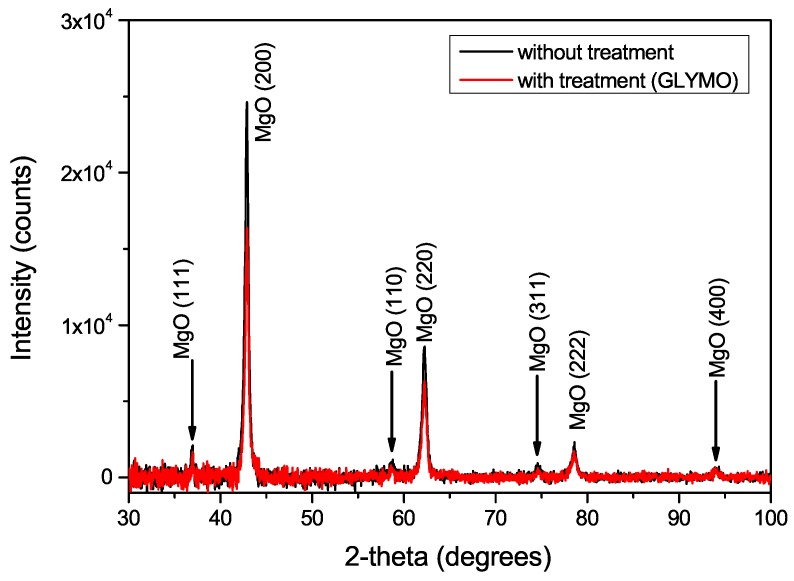
X-ray diagram of treated and untreated MgO nanoparticles.

In this case, also the X-ray diffraction analyse has been performed for confirmation of our uttered assumptions. The X-ray diffraction signals of investigated materials have been deconvolute and diffraction pattern of epoxy matrix has been removed (Figure 10).

There is clearly visible that addition of γ-Glycidoxypropyltrimethoxysilane do not contribute to additional chemical reactions inside the material and the phase structure has not been changed. Hovewer, on the first view, there are a differences between the heights of intensity peaks. The lower intensity peaks may be described by theory of presence of polysiloxane layer on the surface of the magnesium oxide powder [83]. It means that coupling agent occupies a part of volume. This measurement indirectly supports our previous results and statements on the suitability of dielectric response diagnostic methods for evaluation of nanofiller dispersion level.

## 5. Conclusions

This paper presents a few unique results. From the measurement results is visible the effect of dehydration on parameters of complex permittivity. This material evidently contains a significant amount of water molecules in the delivered state. However, these water molecules are with high probability only absorbed or very weakly bonded in the volume of MgO powder if they are released at temperatures below 100 ${}^{\circ }$C. For this reason, it is necessary to dry nanoparticles to remove the surface moisture content before their applications.

Following on the first measurement, the effect of filler loading has been tested for improvement of epoxy matrix properties. The optimal filler loading was set to 1 wt % after dielectric parameters measurement. There are significant changes in investigated parameters, especially in the case of volume resistivity. The increase of volume resistivity is a consequence of the increase in resistance to injection of the charge carriers and their generation in the internal dielectric structure.

The effect of silane coupling agent was also investigated on the optimal filler loading. For the ability to react with different types of matrixes, γ-Glycidoxypropyltrimethoxysilane, has been used. The verification of improvement of dielectric parameters were also carried out. The main reason, why the parameters were improved, are due to a change in the degree of crosslinking due to the reaction of polymeric groups which react with the coupling agent molecules on the nanoparticle surface and form linear polymer chains in the interphase region. Also due to higher degree of dispersion of the filler in the matrix and also to an increase in the energy levels of the electron traps.

Two measurement and evaluation techniques (RRCs, VR) have been taken together in this paper. The possibility of interconnection between these two different techniques is clearly visible from the experimental results. In both cases, the effect of surface treatment of nanofiller was observed on the dielectric response. From this point of view, this methodology can be used not only for evaluation of dielectric parameters, such as conductivity or relaxation time of polarization mechanisms. According to presented results and previous presumptions, these methods may be used for observation of the proper dispersion of nanofiller in a polymer base.

## Figures and Tables

**Table 1:** Selected values of dielectric constant and loss factor for the industrial frequency of 50 Hz.

*T* (${}^{\circ }$C)	$\epsilon^{\prime }$ (-)	$\epsilon^{\prime \prime }$ (-)
25	7.77 (5.80)	1.55 (0.205)
50	8.43 (5.67)	2.68 (0.0801)
75	9.56 (5.57)	4.90 (0.0390)
100	8.65 (5.51)	3.86 (0.0220)
125	7.25 (5.46)	1.90 (0.0145)

Numbers before brackets are values for heating and in brackets are values for cooling.

**Table 2:** Silane coupling agents characterizations [65,66,67].

Coupling Agent	Linear Formula
Trichlorovinylsilane	H_2_C=CHSiCl_3_
Triethoxyvinylsilane	H_2_C=CHSi(OC_2_H_5_)_3_
γ-Glycidoxypropyltrimethoxysilane	C_9_H_20_O_5_Si
γ-Aminopropyltrimethoxysilane	H_2_N(CH_2_)_3_Si(OCH_3_)_3_
[β-(3,4-Epoxycyclohexyl)-ethyl]trimethoxysilane	C_14_H_28_O_4_Si
γ-Mercaptopropyltrimethoxysilane	HS(CH_2_)_3_Si(OCH_3_)_3_

**Table 3:** Comparison of selected parameters after surface treatment.

Sample	Dissipation Factor	Relative Permittivity	Volume Resistivity	Dielectric Strength
Pure resin	0.0033	2.95	6.28 × 10^12^	37 kV/mm
Resin + MgO	0.0041	3.43	5.01 × 10^13^	42.3 kV/mm
Resin + MgO + GLYMO	0.0036	3.15	7.14 × 10^14^	43.1 kV/mm

**Table 4:** Results of voltage response measurement.

Sample	*S_d_*(V/s)	*S_r_*(V/s)
Pure resin	5.26	27.69
Resin + MgO	3.20	16.48
Resin + MgO + GLYMO	2.25	15.33
